# Antimicrobial and Antioxidant Activity of Zinc‐Modified Coconut Husk Biochar

**DOI:** 10.1155/ijbm/1473366

**Published:** 2026-10-05

**Authors:** Ralph Kwakye, Bright Yaw Vigbedor, Albert Aniagyei, Bernard Owusu Asimeng, Justice Wiston Amstrong Jonathan, Boniface Yeboah Antwi, Frimpong Acheampong, David Neglo, Lumor Ernest Kofi

**Affiliations:** ^1^ Basic Science Department School of Basic and Biomedical Sciences University of Health and Allied Sciences Ho Ghana uhas.edu.gh; ^2^ Biomaterials Engineering Department School of Engineering Sciences University of Ghana Legon Ghana ug.edu.gh; ^3^ Institute of Industrial Research Council for Scientific and Industrial Research Legon Ghana csir.co.za

**Keywords:** antimicrobial, biochar, coconut husk, environmental, microorganisms

## Abstract

This study aimed to develop a low‐cost, multifunctional material with both antimicrobial and antioxidant properties by modifying zinc‐derived biochar (BC) from coconut husks. The coconut husk BC was produced through oxygen‐limited pyrolysis at 450°C and was subsequently modified using a zinc nitrate ion‐exchange treatment to create zinc‐modified biochar (Zn‐BC). Characterization techniques such as X‐ray diffraction, scanning electron microscopy, Fourier‐transform infrared spectroscopy, and Raman spectroscopy demonstrated the retention of the carbonaceous BC matrix along with zinc‐containing crystalline domains identified as ZnO. While unmodified BC showed no detectable antimicrobial activity within the tested range (minimum inhibitory concentration [MIC] > 1 mg·mL^−1^), Zn‐BC inhibited all tested bacterial and fungal strains at an MIC of 0.5 mg·mL^−1^. Zn‐BC exhibited enhanced radical‐scavenging activity compared to BC, achieving 67.0 ± 1.5% inhibition of DPPH radicals at 5 mg·mL^−1^ and 57.6 ± 5.2% inhibition of ABTS radicals at 25 mg·mL^−1^. Integration of waste‐derived coconut husk BC with zinc modification results in a material that combines broad‐spectrum antimicrobial activity with measurable antioxidant properties. These findings indicate the need for further evaluation of Zn‐BC in water treatment, environmental applications, and the development of antimicrobial surfaces.

## 1. Introduction

Antimicrobial resistance (AMR) is a significant challenge for global health and the environment.

Traditional medications are losing effectiveness, necessitating new strategies to combat pathogenic microorganisms without worsening resistance [1, 2]. This problem is especially severe in environments like water and soil, where human activities, such as agriculture, mining, and industrial waste discharge, promote microbial adaptation [3, 4]. Heavy metals in these environments contribute to mechanisms that accelerate resistance [5]. To address these challenges, there is an increasing emphasis on developing multifunctional materials that offer both antimicrobial and antioxidant properties. Silver‐based materials have demonstrated effective antimicrobial activity in a variety of composite systems [6]. Biochar (BC)‐supported silver nanoparticle systems, including those from fish scales, exhibit strong bactericidal activity and environmental remediation capabilities, such as dye adsorption [6]. Similarly, BC‐based silver nanoparticles in sodium alginate–tannic acid gel beads have shown high antibacterial efficiency [7]. In polymeric systems, silver nanoparticles embedded in PVA–chitosan composite films demonstrate enhanced antimicrobial properties [6]. Nanocellulose‐reinforced PVA films with silver nanoparticles and plant extracts show improved mechanical strength and significant antimicrobial activity [8]. Despite their effectiveness, silver‐based materials are limited by high costs and environmental persistence. Copper‐based alternatives also exhibit antimicrobial activity through redox cycling and reactive oxygen species (ROS) generation. PVA–chitosan gels with plant extracts and copper nanoparticles exhibit synergistic antimicrobial and wound‐healing properties [9], though copper systems often suffer from oxidative instability at higher concentrations.

Zinc‐based materials effectively combine antimicrobial properties with improved biocompatibility [10, 11]. Zinc’s antimicrobial effects result from the release of Zn^2+^ ions, membrane disruption, and ROS generation, targeting both Gram‐positive and Gram‐negative organisms while reducing resistance [10, 12, 13]. Hybrid systems such as ZnS and Zn–MOF composites show improved performance due to their adjustable porous structures [14]. The abundance of zinc and its environmental compatibility make it sustainable for zinc‐based antimicrobial and antioxidant applications.

BC is a material with high carbon content produced from biomass pyrolysis. It is increasingly used in adsorption, catalysis, and antimicrobial applications [15, 16]. The pores and functional groups containing oxygen in BC enhance its interactions with metal species [17]. Recent research shows that BC‐based composites can be engineered for multifunctional performance [6, 7]. BC can also be modified with active components to boost functionality. For example, chlorophyll or iron‐modified water chestnut shell BC exhibits improved catalytic and antimicrobial properties. Magnetic BC and sodium alginate nanocomposites combine antimicrobial activity and pollutant adsorption with enhanced stability [18]. Lignin‐rich alkaline black liquor‐derived BC composites offer sustainable options with promising antioxidant and antimicrobial performance [19]. The choice of feedstock is crucial. Coconut husk can produce BC with high porosity and stability, making it ideal for creating metal‐modified BC systems with enhanced biological functionality.

This study aimed to develop zinc‐modified coconut husk biochar (Zn‐BC) and investigate the structural changes resulting from zinc modification. The antimicrobial and antioxidant activities of Zn‐BC were evaluated and compared to the unmodified BC. The research explored whether a simple zinc‐loading process could convert a common agricultural byproduct into a multifunctional material. The relationships between structure and properties, as well as various antimicrobial and antioxidant activities within a single Zn‐BC system derived from coconut husks, were assessed. Unlike expensive silver composites or complex metal‐organic systems, this method utilizes a readily available biomass precursor and a simple aqueous modification process.

## 2. Materials and Methods

### 2.1. Materials

Coconut husks were collected from Sokode, Volta Region, Ghana (approximately 5.9406° N, 0.6394° E), washed thoroughly with distilled water to remove adhering soil and debris, and air‐dried to constant mass. Zinc nitrate hexahydrate (Zn(NO_3_)_2_·6H_2_O; analytical grade, ≥ 96% purity) was used as the zinc precursor. The microorganisms used in the test *were Staphylococcus aureus* (*S. aureus*) (NCTC 29212), *Salmonella enterica* serovar Typhi (*S. Typhi*) (ATCC 6539), *Escherichia coli* (*E. coli*) (ATCC 25922), *Klebsiella pneumoniae* (*K. pneumoniae*) (NCTC 13440), *Candida albicans* (*C. albicans*) (ATCC 90028), and *Aspergillus niger* (*A. niger*) (clinical isolate). Their separate colonies were subcultured on nutrient agar and Sabouraud dextrose agar (SDA) before incubation at 37°C for 48 h to ensure their purity [20]. Methanol, 2,2‐diphenyl‐1‐picrylhydrazyl (DPPH), 2,2′‐azino‐bis (3 ethylbenzothiazoline‐6‐sulfonic acid) (ABTS), and other analytical‐grade reagents were obtained from the Cancer Laboratory, School of Basic and Biomedical Sciences, University of Health and Allied Sciences, Ho, Ghana.

### 2.2. BC Synthesis and Zinc Modification

The dried coconut husks were cut into approximately 2–5 cm pieces to promote uniform heat transfer. Pyrolysis was conducted in a custom‐fabricated airtight steel kiln under oxygen‐limited conditions. The temperature was increased at approximately 10°C·min^−1^–450°C and maintained for 40 min. The kiln was then allowed to cool naturally to room temperature before opening to minimize oxidation of the char. The resulting BC was ground using a ceramic mortar and pestle and sieved to obtain particles of 0.75 mm. BC yield was calculated gravimetrically using the equation:

(1) Yield%=Mass of Biochar recoveredMass of dry feedstock×100.

Zn‐BC was prepared by an aqueous zinc‐loading treatment. Sieved BC (3.0 g) was suspended in 70 mL of 0.5 M Zn(NO_3_)_2_·6H_2_O solution and stirred continuously at room temperature for 6 h. The suspension was vacuum‐filtered, and the recovered solid was washed three times with distilled water to remove loosely associated zinc species. The material was oven‐dried at 100°C for 12 h and stored in an airtight container until characterization and biological testing. The preparation process of Zn‐BC, including pyrolysis, zinc incorporation, and application, is illustrated in Scheme 1.

**SCHEME 1:**
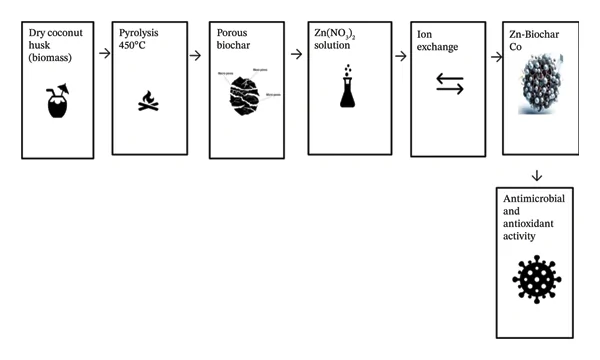
Schematic illustration of the preparation of zinc‐modified coconut husk biochar (Zn‐BC) and its subsequent applications in antimicrobial and antioxidant activities.

### 2.3. BC Characterization

Thermogravimetric (TG) analysis of the BC was conducted using 5–10 mg of a finely ground sample. The sample was held for 10 min and then heated from ambient temperature to 105°C at 10°C per minute. The temperature was then increased to 800°C at the same heating rate under an inert nitrogen atmosphere. At 800°C, the atmosphere was switched from nitrogen to air, and the sample was maintained until a constant mass was achieved. The resulting TG and derivative thermogravimetric (DTG) profiles were analyzed to assess the thermal stability and decomposition behavior of the BC.

Fourier‐transform infrared (FTIR) spectra were recorded over 4000–400 cm^−1^ to identify major surface functional groups and changes associated with zinc modification. Raman spectra were acquired using the available 532/785 nm excitation system over 800–2600 cm^−1^ to evaluate carbon structural order. X‐ray diffraction (XRD) patterns were recorded using Cu Kα radiation (*λ* = 1.5406 Å) over the measured 2*θ* range with a step size of 0.02° to assess phase composition and crystallinity.

The surface morphology was examined using scanning electron microscopy (SEM) at an accelerating voltage of 5–10 kV. A ZEISS EVO 10 SEM was employed to analyze the surface morphology and microstructural characteristics of the BC. Dried BC samples were mounted on aluminum stubs using conductive carbon adhesive tape and, when necessary, were sputter‐coated with a thin layer of a conductive material. SEM micrographs were captured under high‐vacuum conditions at an accelerating voltage of 10 kV and a working distance of approximately 8–12 mm, employing various magnifications. The resulting micrographs were used to assess the surface morphology and structural characteristics of the BC.

### 2.4. Antimicrobial Tests

#### 2.4.1. Minimum Inhibitory Concentration (MIC)

MICs of BC and Zn‐BC were determined by broth microdilution in 96‐well microplates using standard susceptibility‐testing principles [21–23]. A 2 mg·mL^−1^ stock dispersion of each material was prepared in sterile distilled water and serially diluted to produce test concentrations within 0.01–1.0 mg·mL^−1^. Double‐strength Mueller–Hinton broth was used for bacterial assays (Figure 1). Microbial inocula were standardized to 0.5 McFarland before addition. Growth controls contained broth plus organism without test material, whereas sterility controls contained broth without organism. Tetracycline and nystatin served as reference antibacterial and antifungal agents, respectively. After incubation at 37°C for 48 h, 0.1% (w/v) tetrazolium chloride (TTC) was used as a viability indicator. The MIC was defined as the lowest concentration showing no color change consistent with detectable microbial growth. The assay was performed in three independent replicates.

**FIGURE 1:**
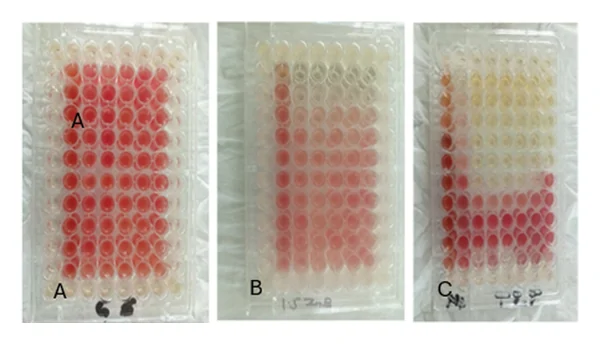
Representative 96‐well microdilution assay plates for (A) BC, (B) Zn‐BC, and (C) controls. TTC color development indicates microbial metabolic activity.

#### 2.4.2. Minimum Bactericidal (MBC) and Fungicidal Concentration (MFC)

MBCs and MFCs were determined by subculturing aliquots from wells showing no visible growth onto appropriate drug‐free agar. Bacterial isolates were subcultured on nutrient agar, while fungal isolates were subcultured on SDA. The plates were subsequently incubated at 37°C for 48 h. The MBC or MFC was defined as the lowest tested concentration that produced no visible colony growth after subculture [24].

The activity ratio was calculated as *R* = MBC/MIC for bacteria or *R* = MFC/MIC for fungi; *R* ≤ 4 was interpreted as bactericidal/fungicidal and *R* > 4 as bacteriostatic/fungistatic.

### 2.5. Antioxidant Activity

#### 2.5.1. DPPH Radical Scavenging Activity

A 0.1 mM DPPH working solution was prepared in ethanol. For each assay, 40 μL of sample was mixed with 160 μL of DPPH solution in a 96‐well microplate to obtain final sample concentrations of 5, 10, 15, 20, and 25 mg·mL^−1^. The plate was incubated for 30 min at room temperature, and absorbance was measured at 517 nm. DPPH solution without sample served as the negative control, and ascorbic acid at the corresponding concentrations served as the positive control. All measurements were performed in triplicate (*n* = 3). Percentage inhibition was calculated as

(2) % inhibition=Ac−At/Ac×100,

where *A*_*c*_ is the absorbance of the control and *A*_*t*_ is the absorbance of the extract.

#### 2.5.2. ABTS Radical Scavenging Activity

The ABTS radical cation (ABTS•^+^) was generated by mixing 7 mM ABTS with 2.4 mM potassium persulfate and incubating the mixture in the dark at room temperature for 12 h. Before use, the ABTS•^+^ solution was diluted with ethanol to achieve an absorbance of approximately 0.70 at 734 nm.

For the assay, 40 μL of the sample was mixed with 160 μL of the ABTS•^+^ working solution, resulting in final concentrations of 5, 10, 15, 20, and 25 mg·mL^−1^. After allowing the mixtures to sit at room temperature for 30 min, absorbance was measured at 734 nm. The ABTS•^+^ solution without the sample served as the negative control, while ascorbic acid was used as the positive control. All measurements were conducted in triplicate (*n* = 3). The percentage inhibition was calculated as follows:

(3) % inhibition=Ac−At/Ac×100,

where *A*_*c*_ is the absorbance of the control and *A*_*t*_ is the absorbance of the extract.

### 2.6. Statistical Analysis

Antioxidant results are presented as mean ± standard deviation (SD) from triplicate measurements (*n* = 3). Two‐way analysis of variance (ANOVA) parametric was used to examine the effects of material type and concentration and their interaction. Where nonconfirmation was required, Kruskal–Wallis tests followed by Dunn’s post hoc comparisons with Bonferroni correction were used. For antimicrobial MIC data, nonparametric comparisons were used because the response values were discrete dilution endpoints and included censored values (> 1 mg·mL^−1^). Statistical significance was set at *p* < 0.05.

## 3. Results and Discussion

### 3.1. BC Yield

Coconut husk was pyrolyzed at 450°C for 40 min, yielding 50.00 g of BC from 120 g of dry feedstock. This corresponds to a yield of 41.60% (w/w) (Table 1). The relatively high char yield is attributed to the lignocellulosic composition of coconut husk and its lignin content, which favor the retention of solid carbon during slow pyrolysis [15, 25–27]. The oxygen‐limited conditions and moderate heating rate help minimize oxidative losses and promote secondary condensation reactions.

**TABLE 1:** Biochar yield from coconut husk.

Type of feedstock	Weight of feedstock (g)	Weight of biochar (g)	Percentage of yield (% w/w)
Coconut husk	120	50.00	41.67

### 3.2. Thermogravimetric Analysis (TGA–DTG) of Coconut Husk BC

The thermal decomposition and structural stability of coconut husk‐derived BC were analyzed using TGA and DTG. From Figure 2, the TGA curve showed a multistage degradation pattern typical of lignocellulosic biomass chars. An initial weight loss of 3%–8% below 150°C was due to the removal of moisture and volatile compounds, indicating effective dehydration during pyrolysis and a limited presence of hygroscopic functional groups in the moderately carbonized material. [25]. The temperature range of 200°C–380°C corresponds to the decomposition of residual organic components, primarily hemicellulose and cellulose, that remain after pyrolysis. The DTG curve shows a peak around 300°C–330°C, indicating the maximum mass loss due to the cleavage of C‐O, C‐C, and glycosidic linkages. This peak suggests that even after pyrolysis at 450°C, some thermally unstable components are still present in the carbon matrix, a common finding in slow pyrolysis BC [27]. A degradation region from 400°C to 650°C corresponds to the gradual decomposition of complex lignin structures and rearrangement of aromatic carbon networks. The DTG signal is less intense and broader in this region, indicating a slower degradation rate and the formation of stable polyaromatic structures, which contribute to BC’s long‐term environmental persistence [15].

**FIGURE 2:**
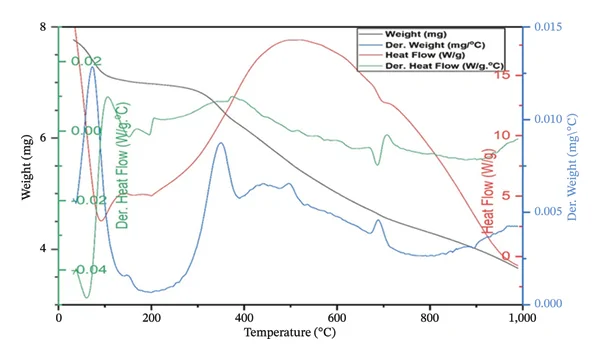
TGA–DTG curves of biochar.

At temperatures above 650°C–700°C, the TGA curve levels off, showing minimal mass loss. A residual mass of approximately 60%–70% indicates fixed carbon and inorganic minerals like silica and calcium ash, confirming significant carbonization and structural condensation. This behavior aligns with previous findings on coconut husk BC, where high lignin content enhances char stability and yield [17, 26]. The lack of sharp or multiple DTG peaks at higher temperatures indicates that the BC is primarily composed of condensed aromatic carbon, rather than thermally labile functional groups. This structural maturity is essential for applications requiring chemical and thermal stability, such as adsorption and catalysis. The observed thermal stability makes this BC suitable for zinc modification, ensuring that zinc incorporation does not compromise its structure. In addition to this, the residual oxygen‐containing groups, as shown in the FTIR analysis, combined with a stable aromatic framework, strike a balance between reactivity and durability [28, 29].

### 3.3. XRD Analysis of BC and Zn‐BC

The XRD pattern of BC was dominated by a broad diffuse feature in the approximately 15°–30° 2*θ* region, consistent with a largely disordered carbonaceous matrix (Figure 3). Sharper reflections superimposed on this background were assigned to mineral phases naturally retained from the biomass ash. The prominent reflection near 26.6° 2*θ* is consistent with quartz (SiO_2_; PDF 46‐1045), while a feature near 29.4° 2*θ* may be associated with calcite (CaCO_3_; PDF 05‐0586).

**FIGURE 3:**
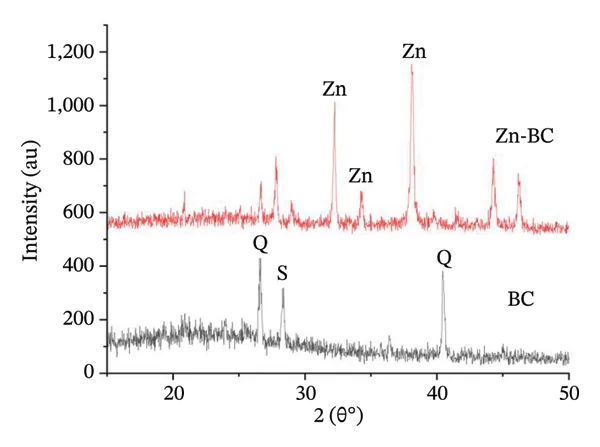
XRD patterns of BC and Zn‐BC. The broad carbonaceous background is retained after zinc modification, while additional reflections in Zn‐BC are consistent with crystalline ZnO domains.

After the zinc modification, Zn‐BC retained the broad carbonaceous background but displayed additional reflections near 2*θ* = 31.7°, 34.4°, and 36.2°, which are consistent with the (100), (002), and (101) planes of hexagonal ZnO (PDF 36‐1451). The broadened reflections are compatible with relatively small crystalline domains. The XRD data therefore support the formation of zinc‐containing crystalline domains assigned to ZnO within or on the BC matrix.

### 3.4. SEM Analysis of BC and Zn‐BC

The SEM micrographs revealed significant morphological differences between raw coconut husk BC and Zn‐BC (Figure 4). The raw BC has a highly porous, irregular structure with interconnected macropores, while the Zn‐BC displays a more compact structure, with pores partially filled and coated by zinc deposits. These morphological changes are consistent with zinc incorporation into the BC matrix and may contribute to the enhanced antimicrobial and antioxidant activities observed for Zn‐BC. The granular deposits observed in Zn‐BC (Figure 4B) may serve as localized antimicrobial sites [28, 29]. Surface zinc species can directly kill bacteria by releasing Zn^2+^ ions and promoting ROS production, resulting in membrane disruption and cell damage [11, 12].

**FIGURE 4:**
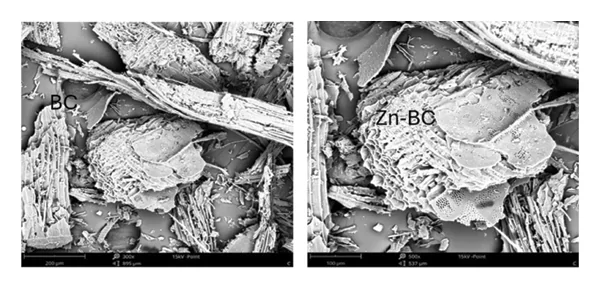
SEM images of unmodified biochar (BC) and zinc‐modified biochar (Zn‐BC).

### 3.5. FTIR Analysis of BC and Zn‐BC

The FTIR spectrum of BC, Figure 5 shows key absorption bands typical of biomass‐derived carbon materials. A broad band at 3400–3430 cm^−1^ indicates O‐H stretching vibrations of hydroxyl groups from phenolic, alcoholic, and carboxylic functionalities, which enhance surface reactivity and metal‐binding capacity [15, 17]. Absorption bands between 2920 and 2850 cm^−1^ indicate residual alkyl structures from incompletely decomposed lignocellulosic components [20]. Prominent bands at 1700–1725 cm^−1^ and 1580–1610 cm^−1^ are attributed to C=O stretching of carboxyl, ketone, and quinone groups, along with aromatic C=C stretching, indicating the formation of condensed aromatic structures. Bands between 1200 and 1050 cm^−1^ indicate C‐O stretching, confirming oxygen‐containing groups on the BC surface. Figure 5 shows that the FTIR spectrum of Zn‐BC reveals changes in band intensity and position. The attenuation and slight shifting of the O‐H (3400 cm^−1^) and C=O (1700 cm^−1^) bands suggest these functional groups are involved in zinc coordination. This indicates that Zn^2+^ ions interact chemically with surface oxygen functionalities rather than being weakly adsorbed [17, 28]. New low‐wavenumber bands at 430–520 cm^−1^ indicate Zn‐O stretching vibrations, confirming the formation of zinc‐oxygen surface complexes [10, 11]. The FTIR findings support SEM observations of uniform zinc dispersion and XRD identification of zinc‐based crystalline domains, indicating that zinc incorporation occurs through surface binding and formation of crystalline zinc‐containing domains. The stabilization of zinc by oxygen‐rich functional groups is crucial for preventing aggregation, which may influence the persistence of the material’s biological activity. The zinc‐oxygen complexes identified by FTIR are essential for the antimicrobial and antioxidant behaviors of Zn‐BC. The BC matrix retains the aromatic carbon, and oxygenated groups scavenge excess radicals, enhancing antioxidant activity.

**FIGURE 5:**
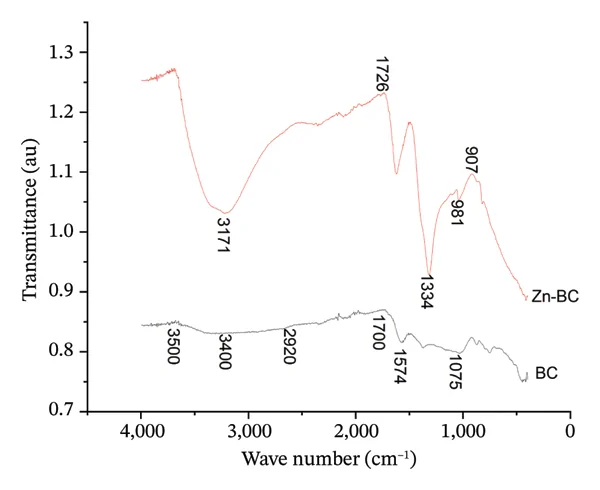
FTIR spectra of BC and Zn‐BC, showing changes in oxygen‐containing functional groups after zinc modification.

### 3.6. Raman Spectroscopic Analysis of BC and Zn‐BC

Raman spectra of both BC and Zn‐BC displayed the characteristic D and G bands typical of disordered carbon materials (see Figure 6) [30, 31]. The reported ratio of the intensity of the D band to the G band (I_D/I_G) decreased from 1.45 for BC to 1.20 for Zn‐BC. This reduction suggests a change in the balance between defect‐associated and graphitic carbon domains following the zinc treatment.

**FIGURE 6:**
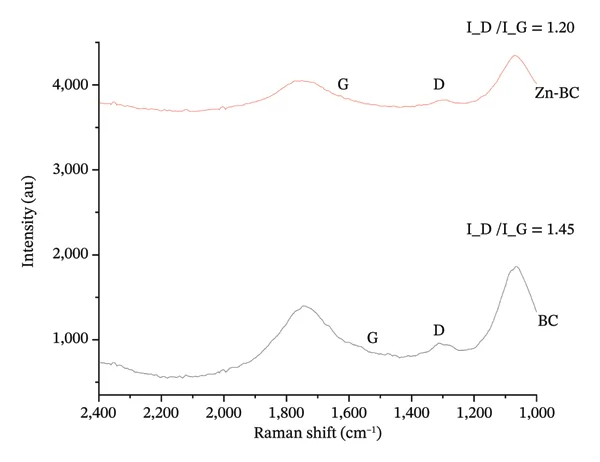
Raman spectra of BC and Zn‐BC.

The combined results from Raman, FTIR, XRD, and SEM analyses indicate that the zinc modification altered both the surface chemistry and structural characteristics of coconut husk BC while preserving its carbonaceous framework. These modifications may explain the enhanced biological activity observed in Zn‐BC. Possible mechanisms for this increased activity include Zn^2+^‐mediated enzyme and membrane disruption, as well as ROS‐related oxidative stress, which have been widely reported for zinc‐containing antimicrobial materials [10–13].

### 3.7. Antimicrobial Activity

BC showed no detectable antimicrobial activity within the tested concentration range (MIC > 1 mg·mL^−1^), whereas Zn‐BC inhibited all six tested microorganisms at 0.5 mg·mL^−1^ (Table 2). The inhibited organisms were specifically identified *as E. coli, K. pneumonia, S. aureus, S. Typhi, C. albicans,* and *A. niger*. Antimicrobial effect was observed against both Gram‐negative and Gram‐positive bacteria as well as yeast and filamentous fungus. The contrast between BC and Zn‐BC indicates that zinc modification was the principal factor associated with the observed antimicrobial activity. The antimicrobial action of Zn‐BC may involve several complementary pathways reported for zinc‐containing materials, including release of bioavailable Zn^2+^, disruption of membrane‐associated processes, and ROS‐mediated damage to cellular components [10–13]. The observed MBC/MIC or MFC/MIC ratios further indicate organism‐dependent killing behavior. Zn‐BC was bactericidal against the tested bacterial strains according to the prespecified *R* ≤ 4 criterion and showed fungicidal activity against the tested fungal organisms within the measured range. The antimicrobial trends observed in Table 2 correlate strongly with the structural and morphological characteristics by SEM, FTIR, RS, and XRD analyses.

**TABLE 2:** Minimum inhibitory concentration (MIC), minimum bactericidal concentration (MBC), minimum fungicidal concentration (MFC), and activity ratio (*R*) of BC, Zn‐BC, tetracycline, and nystatin against the tested microorganisms.

Organisms	BC			Zn‐BC			Tetracycline		
	MIC (mg mL^−1^)	MBC (mg mL^−1^)	*R*	MIC (mg mL^−1^)	MBC (mg mL^−1^)	*R*	MIC (mg mL^−1^)	MBC (mg mL^−1^)	*R*
Bacteria									
*E. coli*	> 1	> 1	> 4 (bs)	0.5	0.5	1 (bc)	0.03	0.03	1
*K. pneumoniae*	> 1	> 1	> 4 (bs)	0.5	0.5	1 (bc)	0.02	0.02	1 (bc)
*S. aureus*	> 1	> 1	> 4 (bs)	0.5	1	2 (bc)	0.0156	0.063	4 (bc)
*S. Typhi*	> 1	> 1	> 4 (bs)	0.5	1	2 (bc)	0.0156	0.063	4 (bc)

**Fungus**	**BC**			**Zn-BC**			**Nystatin**		
	**MIC**	**MFC**	**R**	**MIC**	**MFC**	**R**	**MIC**	**MFC**	**R**

*A. niger*	> 1	> 1	> 4 (fs)	0.5	1	2 (bc)	0.5	0.5	1 (fc)
*C. albicans*	> 1	> 1	> 4 (fs)	0.5	2	4 (bc)	0.125	0.25	2 (fc)

*Note:* MIC, MBC/MFC, and activity classification of BC and Zn‐BC against the tested microorganisms. All concentrations are in mg·mL^−1^. *R* = MBC/MIC for bacteria or MFC/MIC for fungi (*n* = 3). BC, raw biochar; Zn‐BC, zinc‐modified biochar. Activity was classified as bactericidal/fungicidal when *R* ≤ 4 and bacteriostatic/fungistatic when *R* > 4. Here, bc = bactericidal, bs = bacteriostatic, fc = fungicidal, and fs = fungistatic. All concentrations are reported in mg·mL^−1^.

Abbreviations: MBC, minimum bactericidal concentration; MFC, minimum fungicidal concentration; MIC, minimum inhibitory concentration.

Post hoc pairwise comparisons using Dunn’s test with Bonferroni correction showed that Zn‐BC exhibited significantly lower MIC ranks than raw BC across all microbial groups (*p* < 0.001). Reference antimicrobials differed significantly from raw BC (*p* < 0.001). Differences between Zn‐BC and reference antimicrobials were significant for bacterial strains (*p* < 0.01) but not statistically significant for fungal and yeast strains (*p* > 0.05). To further assess organism‐specific responses, Kruskal–Wallis tests were conducted within microbial domains. Significant differences were observed between bacterial and fungal MIC distributions for Zn‐BC (*H* = 6.48, df = 1, *p* = 0.011), indicating a difference in susceptibility between the bacterial and fungal/yeast groups. A Kruskal–Wallis H test was conducted as a nonparametric alternative to assess differences in MIC values among material types, including BC, Zn‐BC, and reference antimicrobials. The results of the test indicated a statistically significant difference in MIC distributions among the treatment groups (*H* = 17.92, degrees of freedom (df) = 2, *p* < 0.001).

### 3.8. Antioxidant Activity

#### 3.8.1. DPPH Radical Scavenging Activity

Zn‐BC demonstrated moderate to high DPPH radical scavenging activity, with mean inhibition values increasing from 53.60% at a concentration of 25 mg·mL^−1^ to 67.02% at 5 mg·mL^−1^ (Figure 7A). This inverse concentration‐response may be attributed to an aggregation effect, where lower concentrations of Zn‐BC allow for more efficient radical scavenging. A similar phenomenon has been observed with metal‐modified BC, where higher concentrations can lead to particle aggregation and masking, which in turn reduces the effective interaction with DPPH radicals [28, 32]. At lower concentrations, Zn‐BC is more evenly distributed, which enables the zinc‐associated redox sites and oxygen‐containing functional groups to interact more effectively with radical species. In contrast, standard BC demonstrated significantly weaker activity in scavenging DPPH radicals, especially at higher concentrations. The inhibition values ranged from 16.62% at 25 mg·mL^−1^ to 61.71% at 5 mg·mL^−1^. Although BC contains phenolic, hydroxyl, and carbonyl groups resulting from biomass pyrolysis, these functionalities alone do not provide strong antioxidant activity, as has been consistently reported for unmodified BC [15, 17]. The improvement observed at lower concentrations is likely due to reduced aggregation and improved surface accessibility rather than increased intrinsic antioxidant strength.

**FIGURE 7:**
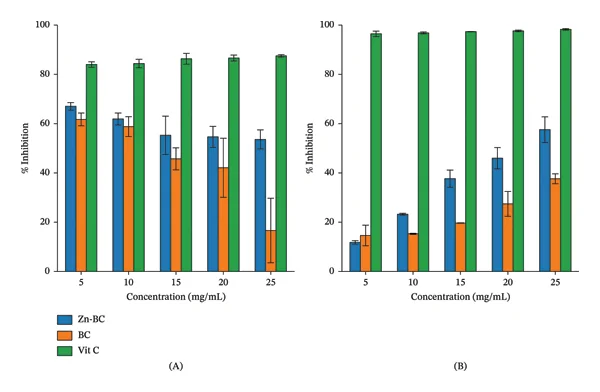
DPPH (A) and ABTS (B) radical‐scavenging activities of BC, Zn‐BC, and ascorbic acid. Data are presented as mean ± SD (*n* = 3).

Ascorbic acid exhibited consistently high DPPH scavenging activity across all tested concentrations, with inhibition values ranging from 83.99% to 87.50%. This behavior is typical of ascorbic acid, which readily donates electrons and hydrogen atoms due to its well‐defined enediol structure. The low variability in the results supports the reliability of the assay and confirms ascorbic acid as a suitable positive control. Similar antioxidant and antimicrobial activities have been reported for plant‐derived flavonoids such as eriodyctiol, highlighting the importance of radical scavenging assays in evaluating multifunctional bioactive materials [33].

The two‐way ANOVA analysis of DPPH radical scavenging activity showed a highly significant effect of material type on antioxidant performance (*F* (2, 30) = 205.00, *p* < 0.001). This indicates that the differences observed are primarily due to variations in composition rather than experimental noise. The large *F*‐value confirms that Zn‐BC exhibits statistically superior activity compared to raw BC, even though there are overlapping error bars at some concentrations. The effect of concentration was also significant (*F* (4, 30) = 14.38, *p* < 0.001), although its influence was weaker than that of material type. This suggests that antioxidant activity is not solely dependent on concentration but is strongly influenced by surface chemistry and zinc incorporation. Additionally, the significant interaction term (*F* (8, 30) = 9.90, *p* < 0.001) indicates that Zn‐BC responds differently to changes in concentration compared to BC. This explains the observed inverse trend, where Zn‐BC demonstrates higher activity at lower concentrations, likely due to reduced aggregation and enhanced exposure of active sites.

Nonparametric Kruskal–Wallis testing corroborated the ANOVA results (*H* = 41.39, *p* < 0.001), confirming robust differences among sample‐concentration groups. Dunn’s post hoc analysis with Bonferroni correction revealed that ascorbic acid differed significantly from both Zn‐BC and BC across nearly all concentrations, while Zn‐BC consistently ranked higher than BC. This pattern indicates a systematic material‐driven enhancement rather than isolated concentration effects.

#### 3.8.2. ABTS Radical Scavenging Activity

Zn‐BC exhibited its highest ABTS scavenging activity at the highest concentration, as shown in Figure 7B, with a mean inhibition of 57.55% at 25 mg·mL^−1^. This activity steadily decreased to 11.80% at 5 mg·mL^−1^. The observed concentration‐response relationship indicates that the ability of Zn‐BC to scavenge ABTS radicals is influenced more by the total number of available redox‐active sites than by surface accessibility alone [34]. At higher concentrations, the increased density of zinc‐associated electron‐transfer centers significantly enhances overall scavenging efficiency. Similar trends have been reported for zinc and metal‐loaded BC, where the bulk redox capacity primarily governs ABTS scavenging activity [29, 35].

BC showed lower ABTS scavenging activity, with inhibition values ranging from 37.62% at a concentration of 25 mg·mL^−1^ to 14.61% at 5 mg·mL^−1^. These findings align with previous studies that characterize raw BC as a weak antioxidant material. The antioxidant activity of BC is primarily influenced by residual aromatic and quinone‐like structures rather than active redox cycling [15, 17].

Ascorbic acid demonstrated near‐complete ABTS radical scavenging, with inhibition values ranging from 99.73% to 99.87%. This confirms its strong reducing power and validates the sensitivity of the ABTS assay. While ascorbic acid is significantly more potent as an antioxidant on a mass basis, it lacks the structural stability and multifunctionality of Zn‐BC. Zn‐BC offers moderate antioxidant activity along with antimicrobial properties, controlled zinc release, and environmental compatibility. This combination makes it particularly appealing for applications in water treatment, environmental remediation, and biologically sensitive systems [28, 29, 35].

In the ABTS assay, the statistical analysis showed an extremely strong effect of material type (*F* (2, 30) = 3327.34, *p* < 0.001). This indicates that the antioxidant performance is primarily influenced by the intrinsic properties of the material. The significantly higher *F*‐value compared to DPPH suggests that ABTS is more sensitive in distinguishing the antioxidant strength among different samples. Concentration also had a substantial impact (*F* (4, 30) = 110.10, *p* < 0.001), confirming a direct concentration‐response relationship for Zn‐BC. Unlike DPPH, ABTS scavenging increased with concentration, indicating that the bulk redox capacity is the dominant mechanism in this case. Additionally, the interaction effect (*F* (8, 30) = 33.42, *p* < 0.001) further confirms that Zn‐BC displays a unique response pattern, demonstrating its dual redox behavior, which involves both ROS generation and radical scavenging.

Kruskal–Wallis analysis confirmed significant group differences (*H* = 43.16, *p* < 0.001). Dunn’s post hoc test showed that ascorbic acid was significantly different from both Zn‐BC and BC, while Zn‐BC consistently ranked above BC, reinforcing the conclusion that zinc modification markedly improves BC antioxidant performance.

In both the DPPH and ABTS assays, antioxidant activity was consistently ranked as follows: ascorbic acid > Zn‐BC > BC. The analysis of effect sizes indicates that the composition of the material, rather than its concentration alone, is the primary factor influencing radical‐scavenging efficiency. The agreement between parametric (two‐way ANOVA) and nonparametric (Kruskal–Wallis and Dunn) analyses supports the validity of these findings. The superior performance of Zn‐BC compared to BC can be attributed to the presence of zinc‐associated redox‐active sites and enhanced surface reactivity. Additionally, ascorbic acid sets a high standard for antioxidant capacity. These results confirm that modifying BC with zinc is an effective strategy to enhance its antioxidant properties, with consistent performance observed across different radical systems.

A statistical analysis using one‐way ANOVA on raw triplicate data also confirmed that the differences observed among BC, Zn‐BC, and ascorbic acid were highly significant across all concentrations (*p* < 0.001). This indicates that the material composition, rather than concentration alone, is the primary factor influencing antioxidant performance. This finding is consistent with existing literature, which identifies metal incorporation as a key determinant of enhanced redox behavior in BC‐based composites [28, 32].

Despite the relatively large error bars observed in some data points, particularly for pristine BC, statistical analysis revealed that the differences among the tested materials were highly significant. One‐way ANOVA of raw triplicate data showed that both DPPH and ABTS radical‐scavenging activities differed significantly across all concentrations (*p* < 0.001). The larger SDs observed, especially for BC at higher concentrations, can be attributed to the inherent heterogeneity of BC materials and variability in surface‐active sites. BC derived from lignocellulosic biomass typically exhibits irregular porosity and non‐uniform distribution of functional groups, which can lead to inconsistent radical interaction and increased experimental variability [15, 17]. Moreover, at higher concentrations, aggregation effects may decrease the effective surface area available for radical scavenging, which can contribute to variability in the measured inhibition values. Similar observations have been noted in metal‐modified BC systems, where particle clustering impacts the accessibility of redox‐active sites [28, 32]. It is important to note that, despite some variability, the high F‐values and extremely low *p* values indicate that the observed differences between BC, Zn‐BC, and ascorbic acid are statistically significant and not the result of random chance. This suggests that the material composition is the primary factor affecting antioxidant performance.

The proposed, literature‐supported antimicrobial and antioxidant pathways of Zn‐BC are illustrated in Scheme 2.

**SCHEME 2:**
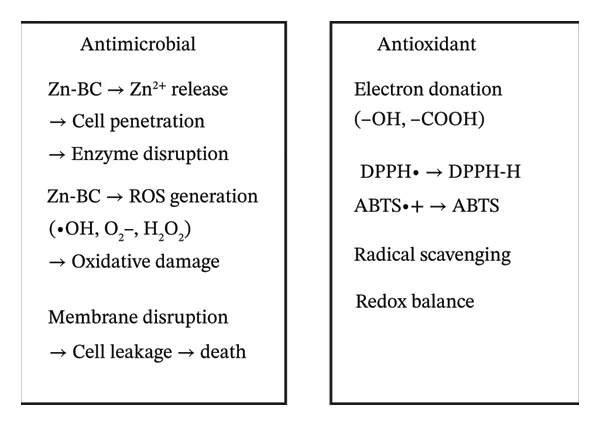
Proposed antimicrobial and antioxidant mechanisms of zinc‐modified biochar (Zn‐BC), showing Zn^2+^ ion release, reactive oxygen species (ROS) generation, membrane disruption, and radical scavenging via electron donation from the biochar matrix.

The principal finding of this study is that a simple zinc modification step transformed coconut husk BC, which exhibited no antimicrobial activity in the tested range, into a multifunctional Zn‐BC with broad‐spectrum inhibitory effects and improved radical‐scavenging capabilities. The relationship between structure and function is evident from XRD reflections indicating ZnO, along with changes observed in FTIR, Raman, and SEM analyses postmodification [10, 13]. These findings support existing literature that shows metal‐containing nanomaterials can combine antimicrobial and antioxidant properties through composition‐dependent surface and redox interactions [36–43].

Recent studies provide valuable context for these findings, as shown in Table 3. For example, plant‐derived extracts have demonstrated both antioxidant and antibacterial activities [36], while CuO nanosheets exhibit combined antibacterial and antioxidant effects [38]. More complex nanomaterial systems, such as antibiotic‐conjugated hydroxyapatite and gold nanoparticles [37], AgTiO_2_ core‐shell nanoparticles [39], MgSCuS nanocomposites [42], TiO_2_‐ciprofloxacin systems [43], and Cu–Ag bimetallic nanoparticles [41], show how careful consideration of composition and interfacial design can enhance antimicrobial performance. In contrast to these engineered nanocomposites, the Zn‐BC approach emphasizes biomass valorization, simple aqueous post‐treatment, and the combination of antimicrobial and moderate antioxidant functions rather than solely focusing on maximum antimicrobial potency.

**TABLE 3:** Comparative analysis of zinc‐modified coconut husk biochar (Zn‐BC) with reported biochar‐based composite systems for antimicrobial applications, highlighting performance, advantages, and limitations.

System	Composition	Antimicrobial performance	Key advantages	Limitations	References
Silver–biochar composite	Biochar + Ag nanoparticles	Very high activity (low MIC)	Rapid bactericidal action	High cost, environmental persistence	[6]
Polymer–AgNP composite film	PVA/chitosan + AgNPs	High antibacterial efficiency	Flexible, biomedical applicability	Expensive, limited scalability	[8]
Copper‐based hydrogel	CuNPs + polymer matrix	Moderate–high activity	Cost‐effective, ROS‐driven activity	Oxidative instability, possible cytotoxicity	[9]
Magnetic biochar composite	Biochar + Fe_3_O_4_	Moderate activity	Reusable, easy separation	Lower antimicrobial potency	[18]
Zn‐based MOF composite	Zn–MOF/ZnS	High antimicrobial activity	Tunable porous structure	Complex synthesis, higher cost	[14]
Zn‐BC (this study)	Coconut husk biochar + Zn	MIC = 0.5 mg·mL^−1^	Sustainable, low‐cost, dual antimicrobial and antioxidant functionality	Moderate potency	This study

The exploration of thiosemicarbazone‐based metal complexes further demonstrates that antioxidant behavior and microbial inhibition can coexist in metal‐containing systems [40]. In this study, Zn‐BC exhibited higher DPPH and ABTS scavenging activity than the unmodified BC, supporting the idea that zinc modification effectively alters the redox‐active surface environment.

## 4. Conclusion

This study shows that zinc modification transforms coconut husk BC into a multifunctional material with antimicrobial and antioxidant properties. XRD, SEM, FTIR, and Raman analyses showed structural and surface changes consistent with incorporation of a zinc‐containing phase, including ZnO‐like crystalline domains, while the carbonaceous BC framework was retained. BC showed no detectable antimicrobial activity within the tested range (MIC > 1 mg·mL^−1^), whereas Zn‐BC inhibited *E. coli, K. pneumoniae, S. aureus, S. Typhi, C. albicans*, and *A. niger* at an MIC of 0.5 mg·mL^−1^. Zn‐BC also showed greater DPPH and ABTS radical‐scavenging activity than BC, although ascorbic acid remained the stronger antioxidant reference. The combination of agricultural‐waste valorization with a simple zinc‐modification strategy produced a material with broad‐spectrum activity against the tested microorganisms and complementary antioxidant properties in one Zn‐BC system.

## 5. Limitations of the Study

Despite its advantages, the Zn‐BC system exhibits lower antimicrobial potency compared to silver‐based composites and only moderate antioxidant performance. Further studies are required to investigate zinc ion release kinetics, long‐term stability, recyclability, and performance under real environmental conditions.

## Author Contributions

Ralph Kwakye: conceptualization, methodology, supervision, investigation, and writing and editing original draft. Bright Yaw Vigbedor: project administration, analysis, methodology, and writing–original draft. Albert Aniagyei: validation, visualization, methodology, and writing. Bernard Owusu Asimeng: conceptualization, methodology, writing, and formal analysis. Justice Wiston Amstrong Jonathan: project administration, writing, analysis, and validation. Boniface Yeboah Antwi: investigation, methodology, validation, and writing. Frimpong Acheampong: analysis, methodology, visualization, and writing. David Neglo: conceptualization, methodology, validation, and writing. Lumor Ernest Kofi: investigation, formal analysis, methodology, and writing–original draft.

## Funding

The authors received no specific external funding for this work.

## Ethics Statement

This study involved material synthesis and in vitro testing of microbial strains only. No human participants, patient cohorts, clinical recruitment, demographic data, cancer staging, or patient treatment histories were involved.

## Conflicts of Interest

The authors declare no conflicts of interest.

## Data Availability

All data generated or analyzed during this study are included in this article and its associated files. Additional raw data may be made available by the corresponding author upon reasonable request.
